# Musings on privacy issues in health research involving disaggregate geographic data about individuals

**DOI:** 10.1186/1476-072X-8-46

**Published:** 2009-07-20

**Authors:** Maged N Kamel Boulos, Andrew J Curtis, Philip AbdelMalik

**Affiliations:** 1 Faculty of Health and Social Work, University of Plymouth, Drake Circus, Plymouth, Devon, PL4 8AA, UK; 2 GIS Research Laboratory, Department of Geography, University of Southern California, Kaprielian Hall (KAP), Room 416, 3620 South Vermont Avenue, Los Angeles, CA 90089-0255, USA

## Abstract

This paper offers a state-of-the-art overview of the intertwined privacy, confidentiality, and security issues that are commonly encountered in health research involving disaggregate geographic data about individuals. Key definitions are provided, along with some examples of actual and potential security and confidentiality breaches and related incidents that captured mainstream media and public interest in recent months and years. The paper then goes on to present a brief survey of the research literature on location privacy/confidentiality concerns and on privacy-preserving solutions in conventional health research and beyond, touching on the emerging privacy issues associated with online consumer geoinformatics and location-based services. The 'missing ring' (in many treatments of the topic) of data security is also discussed. Personal information and privacy legislations in two countries, Canada and the UK, are covered, as well as some examples of recent research projects and events about the subject. Select highlights from a June 2009 URISA (Urban and Regional Information Systems Association) workshop entitled 'Protecting Privacy and Confidentiality of Geographic Data in Health Research' are then presented. The paper concludes by briefly charting the complexity of the domain and the many challenges associated with it, and proposing a novel, 'one stop shop' case-based reasoning framework to streamline the provision of clear and individualised guidance for the design and approval of new research projects (involving geographical identifiers about individuals), including crisp recommendations on which specific privacy-preserving solutions and approaches would be suitable in each case.

## Introduction

### Definitions–the security-confidentiality-privacy triad

In micro-scale geographical analyses involving data about specific individuals, data security, confidentiality and privacy form an intertwined triad. A recent US CDC (Centers for Disease Control and Prevention) foundation course on public health law [1] defines privacy as the "individual's right to control the acquisition, use and disclosure of their identifiable health information". The same course goes on to define confidentiality as the "privacy interests that arise from specific relationships (e.g., doctor/patient, researcher/subject) and corresponding legal and ethical duties", and then describes security as the "technological or administrative safeguards or tools to protect identifiable health information from unwarranted access, use, or disclosure". To explain the relationships between the three terms, the course quotes a key sentence from Ware [2]: "If the security safeguards in an automated system fail or are compromised, a breach of confidentiality can occur and the privacy of data subjects invaded".

### Mainstream media and public interest in the subject

Actual or potential breaches (technological or legal) of data security and confidentiality and the subsequent actual or potential invasions of individuals' privacy are quite commonly reported in mainstream media. For example, in March 2009, the Joseph Rowntree Reform Trust published its 'Database State' report on the legality, safety and effectiveness of the British government's major database systems [3,4]. Of 46 databases assessed in this report, only six were found to have a proper legal basis for any privacy intrusions and were deemed proportionate and necessary in a democratic society. The report authors concluded that two NHS (National Health Service) systems, the Detailed Care Record (DCR) and the Secondary Uses Service (SUS) [5], were almost certainly illegal and that a number of others including the Summary Care Record (SCR) would be legal only with patient consent, but, with the current absence of an effective opt-out, it too was almost certainly illegal.

We also read the story of an anonymous Canadian girl whose death was associated with a prescribed acne drug. She was eventually identified by the media who compared the de-identified prescription data set against obituaries. The comparison helped in narrowing down the search to four possible girls, then by contacting all families the right one was found [6].

High-profile security breaches (e.g., data loss or theft of ill-protected confidential data) are also not uncommon. For example, it was reported in May 2009 that a laptop containing non-encrypted data (names, addresses, dates of birth, employers, national insurance numbers, salary information, and bank details) of 109,000 UK pensioners has been stolen [7]. The data were merely password-protected, and possibly without any appropriate safeguards for data self-destruction in case of brute-force password attacks. It is very easy to find out passwords in a short time using common hardware, e.g., NVIDIA CUDA GPUs (Compute Unified Device Architecture Graphics Processing Units), and readily available software [8], or even to completely bypass the passwords and directly access the underlying non-encrypted data.

In public health worldwide, any public identification of an individual's health status and address, regardless of contagion level or risk, is usually prohibited. But individual privacy rights must also be balanced with legitimate public concerns and interests. The publicly-accessible, online mapping of SARS (Severe Acute Respiratory Syndrome) in Hong Kong a few years ago using disaggregate case data at individual infected building level in near real time was one of the noticeable exceptions to the well-established public health confidentiality rule [9,10].

## Research literature: location privacy concerns and solutions

The biomedical and public health literature on geographic information systems (GIS) and spatio-temporal analyses features a large number of research papers mentioning or addressing location privacy, e.g., [11-28]. A must-read paper (not specifically health-related) dating back to 1994 [29] shows how chronic privacy issues are in GIS research. Some research papers identified privacy as a potential or actual issue of concern (e.g., in reproductive health research [18]; in birth defects surveillance and research [19]; in research relevant to policy on diet, physical activity, and weight [20]; in environmental health research [21]; and in health and social care planning [22]), while others went one step further by suggesting some comprehensive solutions (e.g., [23-26]), workarounds, or frameworks and principles of practice (e.g., [29]) to mitigate or resolve these privacy concerns.

A number of confidentiality-preserving statistical and epidemiological data processing methods (data aggregation and transformations) have been proposed that can be applied to original location data to preserve individuals' privacy while maintaining some acceptable level of data usefulness for geographical analyses. But the use of precise addresses will continue to be needed in many cases to improve data analysis results or make them possible at all. The famous John Snow's map of the 1854 Cholera outbreak in London only solved the problem because the unique locations of individual cases were known [15,30]. There will always be this implicit trade-off between privacy concerns (e.g., easiness of re-identification) and the types and accuracy of the results of geographical health analyses that are possible with a given data set (original, unaltered vs. transformed or aggregated data) [25,27,28]. And that is where software agents can offer a potential solution that preserves the full fidelity of the original data [25].

Moving beyond conventional GIS research and geographical analyses, mobile phones and other electronic gadgets are rapidly gaining location awareness and wireless Web connectivity, thus promising new spatial technology applications and services (e.g., [31-33]), which will yield vast amounts of spatial information and online maps that can even reveal users' whereabouts in real time. These novel spatial tools and services are certainly opening many new useful possibilities, but are not without their challenging security and privacy concerns [34,35].

## The 'missing ring': data security

Data security is relatively under-mentioned in discussions about confidentiality-preserving solutions for location data, despite its key importance in the aforementioned security-confidentiality-privacy triad. Consider the following scenario: a health GIS researcher has legitimate and IRB (institutional review board)-approved access to patient data containing precise geographical identifiers for analysis and reporting purposes, with full patient consent. The reporting is done in ways that do not identify individual patients when posting publicly-accessible/online results and maps. If the reporting must be made at some level of detail or granularity that can potentially identify individual patients, the results are only shared within approved, small teams of users with legitimate access rights and 'need to know'. The whole scenario seems fine as far as the protection of individuals' privacy is concerned. IRB approval has been sought, adequate reporting methods and policies are in place to prevent the disclosure of any confidential data to non-authorised parties, and we even have the patients' explicit consent to conduct the study. However, without appropriate additional security safeguards, there will always be many unmitigated risks of data theft or loss and of unwanted data disclosure to non-authorised or non-authenticated parties, all of which can compromise the privacy of the data subjects. (Ideally, IRBs should be scrutinising the security component as well before granting approvals.)

A carefully blended, purpose-built combination of overlapping security measures is always the solution, depending on the type, sensitivity, value, and risks/costs assessment of the data to be protected. Various types of advanced cryptography, multimodal biometrics, and other methods can be combined, as necessary. Data access can also be controlled or restricted in such a way that two or more persons must be physically present each time and authenticated (e.g., via biometrics) to unlock the data. Security measures cover and include, among other things, ensuring physical building security, using computer security cable locks, using computers with a built-in TPM (Trusted Platform Module) chip for cryptographic functions, performing full disk encryption with TPM (e.g., using BitLocker [36]), implementing brute-force password attack protection (data are automatically erased after a pre-set number of failed access attempts), using hardware/software firewalls and other forms of network security, implementing adequate access policies and authentication [37] (at computer BIOS–Basic Input/Output System level, Operating System-level, and application level), considering Multilevel Security (MLS), using biometrics (e.g., fingerprint readers and facial recognition), using advanced secure USB flash drives with military grade hardware encryption (e.g., [38,39]) instead of ordinary flash drives, keeping detailed data inventories and electronic audit trails of all accesses and transactions, blanking of computer display and machine locking or auto-log-off if a machine is left unattended, and the secure decommissioning and discarding of old equipment and data storage media, e.g., using software utilities like SDelete [40] to prevent the kind of issues described in [41]. Also equally important are staff training and the development of a 'security culture' in the organisation, e.g., guided by ISO/IEC 27002 2005 (formerly ISO/IEC 17799 2005), an ISO (International Organization for Standardization) standard for information security and a code of practice for information security management.

## Personal information and privacy legislations

A discussion on location privacy solutions for health research would be incomplete without reflecting on some of the underlying reasons that necessitate their development. The very notion of privacy is itself a complex fabric of interwoven philosophical and psychosocial threads. Perhaps this is why the associated bureaucratic and legal landscape is as complex as it is – and often blamed for the issue. A large majority of public health professionals consider privacy to be an obstacle to public health; when asked for the underlying reasons, survey respondents in Canada and the UK most commonly identified bureaucracy and legislation [42].

There is no universal legislation to guide and govern the activities of public health professionals, particularly where issues of privacy are concerned. Instead, nations have their own constraining or enabling privacy and data protection laws, with some being such a maze of cross-referenced "legalese" that familiarising oneself with them – let alone gaining a thorough understanding of them – becomes a daunting task. 'Additional file 1' provides a brief compilation and comparison of relevant personal information and privacy legislation in Canada and UK, with particular focus on location and public health as seen and understood by an epidemiologist.

## Some recent research projects and events about the subject

The issues of location privacy were also the subject of GeoPKDD (Geographic Privacy-Aware Knowledge Discovery and Delivery [43]), a three-year EU-funded project that was recently completed in November 2008. GeoPKDD's main research question was 'how to discover useful knowledge about human movement behaviour from mobility data (e.g., location data from mobile phones), while preserving the privacy of the people under observation?' The project attempted to develop new privacy-preserving methods for extracting knowledge from large amounts of raw data about individuals referenced in space and time. GeoPKDD organised the 'First Interdisciplinary Workshop on Mobility, Data Mining and Privacy: Preserving anonymity in geographically referenced data' on 14 February 2008 in Rome, Italy [44].

Another research activity worth mentioning in our context is the proposal by Helen Chen at Agfa HealthCare in Canada and her colleagues at the World Wide Web Consortium–W3C Semantic Web for Health Care and Life Sciences Interest Group (HCLSIG) to explore Semantic Web solutions for patient data security, confidentiality, consent and privacy (in general, i.e., they are not focusing on location privacy, but their proposal is still broadly relevant to our topic). Previously sufficient de-identification techniques can be rendered inadequate because it is now possible to re-identify an identity via inference on the Web. Semantic Web technology is making headway to even more powerful data links, connections and inferences of this type. However, in the healthcare domain, this very success of the technology is putting individuals' privacy at much greater risks. Chen's idea is to develop novel privacy-preserving solutions by harnessing the very same Semantic Web technology that can exacerbate these privacy risks [45].

From 5–8 June 2009, the Urban and Regional Information Systems Association (URISA [46]), a non-profit American association of professionals using GIS and other information technologies to solve challenges in state/provincial and local government agencies and departments, organised its Second GIS in Public Health Conference in Providence, Rhode Island, USA. One of the pre-conference workshops held on the 5th of June 2009 focused on issues related to 'Protecting Privacy and Confidentiality of Geographic Data in Health Research'. Select highlights from this workshop are presented in the remaining part of this article.

## Select highlights from a recent URISA workshop on location privacy in health research

At the 2009 URISA GIS in Public Health Conference, a workshop organised by Ellen Cromley and Andrew Curtis focused on the issue of location privacy in health research. Among the topics covered by panellists and attendees were methods of spatial data protection, the need to "educate" IRBs, challenges facing data owners and custodians wishing to visualise and disseminate data, how published maps continue to violate confidentiality, some general cartographic guidelines and "fixes", and new methods of spatial data masking. In addition, the participants spent considerable time discussing the ethical and legal challenges researchers now face as HIPAA (US Health Insurance Portability and Accountability Act) regulations change, placing more responsibility on the data user (researcher). Although the majority of attendees to the meeting were data owners or custodians, this article is written mainly from the perspective of the data user, especially a social science/geographic information science researcher. As researchers, our usual role is to spatially analyse data, collect new spatial data with health implications, and visualise results in multiple forums, especially academic journals.

In 2006 Curtis *et al*. published a paper in *International Journal of Health Geographics *highlighting the potential for point level data to be reengineered from published maps through a process of digital scanning and georeferencing, even with only limited geographical features [11]. By heads-up digitising these points, coordinates could be used to direct field teams to actual homes. This conceptual approach had previously been impossible to replicate with real data, but by using this case from Hurricane Katrina, the map of mortality locations, and search and rescue markings that actually identified where bodies were found, validation was possible. Concurrent to this article, other reengineering approaches appeared using simulated data and a more systematic approach to identifying homes from a low resolution map [12]. Both papers revealed that published maps, even of low resolution and with limited geographical information, could still be reengineered back to an exact address, or so close to the 'real world' location that even without resorting to use other quasi identifiers, the spatial confidentiality of those being mapped was violated.

As researchers specialising in geographic information, we need to be proactive in setting guidelines for the display of confidential data, in policing our own actions, and in educating those sitting in positions of data power, especially our IRBs. Critics of the presentation usually focus on the data source–"this is a newspaper map so there is no confidentiality violation". However, there have been at least two maps appearing in journals that have also published the same Katrina mortality point locations. But irrespective of this, the real message is, *any point level map can be reengineered back in the same way*. As academics where does our ethical path lie with these secondary sources obtained from the media? We may not legally be violating confidentiality, but does that give us the right to use non-official sources, apply our geospatial skills and create sensitive layers in other outlets?

Now in mid-2009, what has changed? Are maps still being published in academic journals that violate spatial confidentiality? And where are we on the issue of cartographic guidelines? Unfortunately it is still too easy to find similar map violations appearing after 2006. One can find examples of maps with point level mortality locations, pregnancies, at-risk pregnancy programme participants, and people suffering from different respiratory ailments – indeed we challenge the reader to see how many point level health maps they can find. Of particular concern are those sub-disciplines which have just discovered the value of GIS–we cannot expect that confidentiality violation through cartographic design is uppermost in the minds of those effusing over the wonders of buffering.

What else has changed? Paradoxically, the attention currently being paid to geocoding accuracy – which is important from a health research perspective, and which has received considerable attention in *International Journal of Health Geographics *– also has a detrimental side in terms of making published source maps both more accurate and precise. This means the chance of successful reengineering in terms of being closer to the actual address has increased. In effect, this previously unintentional form of masking has been reduced. Secondly, smoothing approaches, such as density surfaces, are being used to preserve confidentiality in maps (and stated as such by the authors). On one hand this is good news in terms of researchers' understanding that there is a confidentiality issue, but on the other hand this quick-fix is problematic due to a reliance on techniques that do not achieve this goal. The combination of window/kernel/filter size, the underlying grid cell resolution, and especially if there is no option for a minimum denominator, may result in "bulls-eyes" for areas of the map with relatively few residential alternatives, otherwise known as the 'geographic area population size' [47]. It should also be remembered that less dense geographies are not necessarily rural; many urban areas also contain physical features (inlets, lakes, even hills) can remove alternative possible locations. By referring to high resolution aerial photography (now found easily in applications such as Google Earth [35]), it is relatively easy to identify the cause of the intensity. On this subject, geospatial Internet applications in general have made the reengineering process even easier for those with and without a working knowledge of GIS.

From a data users' perspective, we are still limited by data being released at an aggregation that is limited for research, the standard for HIPPA being a zip code with 20,000 individuals. A group of Canadian researchers showed that this is an archaic approach and that minimum denominators should vary when taking into account the underlying geography and the number of quasi identifiers [47]. Similar papers written for researchers in other countries, possibly even providing a series of size guidelines for different urban areas, would be invaluable. It would also help the job of IRBs.

And on the subject of IRBs, from our experience there is still a disconnection in terms of understanding exactly where the risks lie in geospatial output and confidentiality. This is understandable given the confusion even amongst geospatial researchers. What would benefit all concerned would be a well-respected body in the field of public health to commission a "guidelines" paper. This could become *the reference *in terms that researchers, IRBs, and even research subjects could understand and cite, along with other existing key texts, such as [48]. These reference guidelines should include clear visual examples of what is not acceptable, including the pitfalls of common "fixes" such as smoothing. They could also provide guidance for appropriate aggregation denominator sizes. This is important as researchers seek IRB approval in the use of mobile geospatial devices for collecting health and built environment data. We cannot expect IRBs to understand where such cartographic risks lie. Finally, language should be included that would help IRBs and be required in any letter gaining subject permission. In other words, *"if we ask for an address (or a street intersection... or a zip code...) this is the only way we will display it on a map"*. This simple approach would mean that IRB, researcher and subject would all have the same understanding of what will happen with these data. (Ellen Cromley has vested considerable time on spatially appropriate language for informed consent as a guideline for IRBs. She disseminated examples of this language at the URISA workshop.)

This 'best practice guide' should be circulated to all journals who publish maps, clearly stating the risks involved in accepting point level maps. At least this would enlighten editors [49] and hopefully force them to ask submitting authors about '*what steps have been taken to preserve confidentiality?*'

Until we have such a universally accepted document, self-policing is the main option, and with this in mind, we have a few issues a researcher should ponder before publishing any map. Most importantly, is a point-level (or smoothed, or small aggregation) map necessary? As a geographer this last statement certainly hurts, but unless a map is really needed to help frame a paper's content, or improve the understanding of the reader regarding a spatial process, and especially if it is not even specifically referred to in the text, then it is better to err on the side of caution.

We fully realise that some point-level maps will still need to be published; it is often easier to explain a spatial process through a graphic, but if this is the case then is the underlying geography needed? If we are overlaying points against output from a spatial analysis, do we need political boundaries or street networks? If geographical references are necessary in the map, then data masking is essential.

There is some good news though, as we have noticed more researchers referencing steps taken to preserve confidentiality during recent presentations.

### Emerging issues

There are three emerging spatial confidentiality topics of concern. The first involves Google Street View [50], an excellent research tool that allows us to "see" areas that are described or mapped in publications. The implication this has for reengineering is the ability to see potential candidates within an area. If we again think of the "bulls eye" effect within a smoothed surface, if this area has been driven by the Google Street View team (and thankfully at this point areas of sparse geography also tend to be the least covered), we could literally view each option within the central pixel until a house match is found. Even with multiple alternatives, it might be possible to spatially prioritise the potential buildings based on characteristics of the health conditions, or other information gleaned from the paper. For example, is the disease more typically associated with a multi-family unit than a single residence?

The second area of concern involves the use of biometric sensors synched to a GPS (Global Positioning System) unit. This field of research offers great potential in terms of linking health outcomes to the fine-scale built environment. However, a fear expressed at the URISA workshop was that output from these devices, usually shown as a series of dots on an aerial photograph, will begin to accompany research papers. Sure enough, within one day of the workshop a new issue of a GIS journal published this exact output. The underlying aerial photograph makes reengineering from the image extremely easy, and the point concentrations from the GPS unit correspond to areas of highest activity, including the home. This is not a good situation, especially when the participants are part of a vulnerable population, such as children.

Finally, we are worried about the current trend by social scientists of including spatial data in their research, especially those who use mixed methods. A mixed method approach combines both qualitative and quantitative data. For example, spatial video data of the recovering neighbourhoods of New Orleans, LA, USA, are currently being collected. These data are extracted from the video as three-dimensional surfaces that can be mapped or analysed for recovery or abandonment. At the same time, videos of the narrative of the neighbourhood participants add further commentary to the surfaces, such as why a building has not been returned to. Many of these comments contain sensitive information such as the health of an owner. If we map this information, others could easily disseminate it through online consumer geoinformatics services like Google Earth and Google Map, and even link it using suitable geo-mashups [51] to other readily available online information about the individuals concerned (e.g., on social networking sites), thus revealing a more detailed picture about them. Do our subjects really know all what could be exposed through such mapping? (But one should also consider the difference between what is *technically possible *and what is *practically likely to happen*, i.e., will there really be someone with the motive, will and ability to do these privacy threatening Web inferencing and mapping exercises in each and every case? A risks-costs-benefits assessment might help in such situations.) Although these situations may not fall foul of any HIPAA standard, nor probably concern an IRB, we are now at a point where changing geospatial technologies must stimulate debate that goes beyond the normal community participatory ethical standards used by researchers [35].

Because of the widespread adoption of GIS-light Internet applications, and cheap and easy-to-use mobile mapping devices (for example, ones which can tag pictures with coordinates), health related spatial confidentiality is now no longer the concern of only geographic information scientists, or even GIS users, but also of a far broader range of academics and other people.

## Conclusion

Although the general public's concerns about privacy in research have sometimes been exaggerated by the scientific community [52] (and by a few vocal privacy advocates in the media, who do not adequately represent the position of the wider masses), we believe there are still many cases where these concerns are real and legitimate, and where data subjects need to be protected (e.g., from identity theft). A 'one-size-fits-all' privacy-preserving solution is unlikely to be successful or to be able to capture and properly address the complex requirements, which might also vary from country to country, of the very many (i) user roles, with different access privileges and 'needs to know' in relation to various input and output data types; (ii) intra and extra-mural data sharing arrangements, especially when data need to be moved across heterogeneous organisations; (iii) governing legislations and policies; (iv) possible forms of data inputs that can be released for research and the associated conditions; (v) health study types and goals, data analysis methods and the data requirements in each case; (vi) possible study outputs/results reporting and publication forms (closed or public); (vii) situation-specific security risks; and (viii) risks-costs-benefits assessment, among other aspects and requirements that are involved in this area of research and need to be considered on a case-by-case basis.

Different privacy-preserving solutions can be applied concurrently or singly on various elements of this complex chain, e.g., on input data prior to release to researchers (e.g., aggregation or transformations) and/or on the research outputs (e.g., access restriction or masking), depending on the specific situation at hand; so a comprehensive, context-aware approach is needed to assist researchers in choosing and applying the right solution(s) in each case.

Kamel Boulos (unpublished research notes, 2008–2009) proposed the development of a case-based reasoning software framework (*cf*. case law) that covers, and continuously "learns from", the growing body of possible and emerging health research scenarios and applications involving precise geographical identifiers about individuals. The goal of such a 'one stop shop' framework would be to streamline the provision of clear and individualised guidance for the design and approval of new research projects, including crisp recommendations on which specific privacy-preserving solution(s) and approach(es) would be suitable in each case. This would spare researchers and IRBs the need to 'reinvent the wheel' with each new study, saving them precious time and efforts spent investigating the same issues every time, and preventing avoidable errors and omissions along the way. This decision framework should ideally have an easy-to-use, wizard-based visual frontend, guiding users throughout the whole process of describing and diagnosing their needs, and proposing (with appropriate explanations/justifications) suitable solutions to address them.

## Competing interests

The authors declare that they have no competing interests.

## Authors' contributions

MNKB conceived and drafted the manuscript, and conducted a mini-survey of the literature on the subject. AJC provided material on URISA's workshop held on 5 June 2009 about location privacy issues in health research. PA contributed material on 'personal information and privacy legislations', including 'Additional file 1'. All authors read and approved the final manuscript.

## Supplementary Material

Additional file 1 A brief compilation and comparison of relevant personal information and privacy legislation in Canada and the UK, with particular focus on location and public health Click here for file

## References

[B1] US CDC Public Health Law 101 Foundational Course for Public Health Practitioners – Unit 6: Privacy and Confidentiality. http://www2a.cdc.gov/phlp/phl101/docs/PHL101-Unit%206%20-%2016Jan09-Secure.ppt.

[B2] Ware W (1993). Lessons for the future: Privacy dimensions of medical record keeping. Proceedings of the Conference on Health Records: Social Needs and Personal Privacy, Sponsored by the Department of Health and Human Services Task Force on Privacy, Office of the Assistant Secretary for Planning and Evaluation and the Agency for HealthCare Privacy and Research: 11–12 February 1993 (Document No PB94-168192).

[B3] Anderson R The devil is in the detail-A case in Finland on the privacy of medical records puts two major NHS systems in legal peril (Smart Healthcare – 1 April 2009). http://www.smarthealthcare.com/anderson-database-01apr09.

[B4] Anderson R, Brown I, Dowty T, Inglesant P, Heath W, Sasse A (2009). Database State.

[B5] Secondary Uses Service (SUS) – NHS Connecting for Health. http://www.connectingforhealth.nhs.uk/systemsandservices/sus.

[B6] Malheiros M Medical data secondary use issues (Privacy Value Networks – 10 June 2009). http://www.pvnets.org/2009/06/medical-data-secondary-use-issues/.

[B7] Pension details of 109,000 stolen (BBC News – 28 May 2009). http://news.bbc.co.uk/1/hi/business/8072524.stm.

[B8] ElcomSoft Distributed Password Recovery Software: High-performance distributed password recovery with NVIDIA GPU acceleration. http://www.elcomsoft.com/edpr.html.

[B9] Kamel Boulos MN (2004). Descriptive review of geographic mapping of severe acute respiratory syndrome (SARS) on the Internet. Int J Health Geogr.

[B10] Woo RB Epidemics, Privacy Rights and Public Concerns: The Hong Kong SARS Experience. Workshop: Globalisation and New Epidemics: Ethics, Security and Policy Making, Organised by European Commission – Science and Society: 22–23 May 2006; Brussels, Belgium.

[B11] Curtis AJ, Mills JW, Leitner M (2006). Spatial confidentiality and GIS: re-engineering mortality locations from published maps about Hurricane Katrina. Int J Health Geogr.

[B12] Brownstein JS, Cassa CA, Kohane IS, Mandl KD (2006). An unsupervised classification method for inferring original case locations from low-resolution disease maps. Int J Health Geogr.

[B13] Cassa CA, Wieland SC, Mandl KD (2008). Re-identification of home addresses from spatial locations anonymized by Gaussian skew. Int J Health Geogr.

[B14] Van Wey LK, Rindfuss RR, Gutmann MP, Entwisle B, Balk DL (2005). Confidentiality and spatially explicit data: concerns and challenges. Proc Natl Acad Sci USA.

[B15] Gutmann M, Witkowski K, Colyer C, O'Rourke JM, McNally J (2008). Providing Spatial Data for Secondary Analysis: Issues and Current Practices relating to Confidentiality. Popul Res Policy Rev.

[B16] Werneck GL (2008). Georeferenced data in epidemiologic research. Cien Saude Colet.

[B17] Cassa CA (2008). Privacy and identifiability in clinical research, personalized medicine, and public health surveillance. PhD thesis.

[B18] Sherman JE, Fetters TL (2007). Confidentiality concerns with mapping survey data in reproductive health research. Stud Fam Plann.

[B19] Siffel C, Strickland MJ, Gardner BR, Kirby RS, Correa A (2006). Role of geographic information systems in birth defects surveillance and research. Birth Defects Res A Clin Mol Teratol.

[B20] Matthews SA, Moudon AV, Daniel M (2009). Work group II: Using Geographic Information Systems for enhancing research relevant to policy on diet, physical activity, and weight. Am J Prev Med.

[B21] Smolders R, Casteleyn L, Joas R, Schoeters G (2008). Human biomonitoring and the INSPIRE directive: spatial data as link for environment and health research. J Toxicol Environ Health B Crit Rev.

[B22] Foley R (2002). Assessing the applicability of GIS in a health and social care setting: planning services for informal carers in East Sussex, England. Soc Sci Med.

[B23] Armstrong MP, Rushton G, Zimmerman DL (1999). Geographically masking health data to preserve confidentiality. Stat Med.

[B24] Cassa CA, Grannis SJ, Overhage JM, Mandl KD (2006). A context-sensitive approach to anonymizing spatial surveillance data: impact on outbreak detection. J Am Med Inform Assoc.

[B25] Kamel Boulos MN, Cai Q, Padget JA, Rushton G (2006). Using software agents to preserve individual health data confidentiality in micro-scale geographical analyses. J Biomed Inform.

[B26] Wieland SC, Cassa CA, Mandl KD, Berger B (2008). Revealing the spatial distribution of a disease while preserving privacy. Proc Natl Acad Sci USA.

[B27] Olson KL, Grannis SJ, Mandl KD (2006). Privacy protection versus cluster detection in spatial epidemiology. Am J Public Health.

[B28] Ozonoff A, Jeffery C, Manjourides J, White LF, Pagano M (2007). Effect of spatial resolution on cluster detection: a simulation study. Int J Health Geogr.

[B29] Snow J On the Mode of Communication of Cholera.

[B30] Onsrud HJ, Johnson JP, Lopez X (1994). Protecting Personal Privacy in Using Geographic Information Systems. Photogrammetric Engineering and Remote Sensing.

[B31] Google Latitude. http://www.google.com/latitude.

[B32] Microsoft Vine. http://www.vine.net/.

[B33] Yahoo! Fire Eagle. http://fireeagle.yahoo.net/.

[B34] Mokbel MF (2007). Privacy in Location-Based Services: State-of-the-Art and Research Directions. Proceedings of the 8th International Conference on Mobile Data Management (MDM'07): 7–11 May 2007; Mannheim, Germany.

[B35] Kamel Boulos MN, Madden M (2009). Chapter 49: Principles and techniques of interactive Web cartography and Internet GIS. Manual of Geographic Information Systems.

[B36] Microsoft Windows BitLocker Drive Encryption. http://technet.microsoft.com/en-us/library/cc766200(WS.10).aspx.

[B37] Securing Sensitive Information with Identity and Access Assurance (RSA/Courion White Paper). http://www.rsa.com/solutions/IA/wp/10292_RSA-Courion_WP_0609.pdf.

[B38] IronKey: Secure USB Flash Drive with Internet Protection Services. https://www.ironkey.com/.

[B39] Integral Crypto Drive. http://www.integralmemory.com/crypto/?gclid=CMW528i5ipsCFU0B4wod7GSUoA.

[B40] SDelete. http://technet.microsoft.com/en-us/sysinternals/bb897443.aspx.

[B41] El Emam K, Neri E, Jonker E (2007). An Evaluation of Personal Health Information Remnants in Second-Hand Personal Computer Disk Drives. J Med Internet Res.

[B42] AbdelMalik P, Kamel Boulos MN, Jones R (2008). The perceived impact of location privacy: a web-based survey of public health perspectives and requirements in the UK and Canada. BMC Public Health.

[B43] GeoPKDD – Geographic Privacy-Aware Knowledge Discovery and Delivery. http://www.geopkdd.eu/.

[B44] First Interdisciplinary Workshop on Mobility, Data Mining and Privacy: Preserving anonymity in geographically referenced data: 14 February 2008; Rome, Italy. http://wiki.kdubiq.org/mobileDMprivacyWorkshop/.

[B45] HCLS Patient Data Security and Privacy. http://esw.w3.org/topic/HCLS/SecurityPrivacy.

[B46] URISA – The Association for GIS Professionals. http://wwww.urisa.org/.

[B47] El Emam K, Brown A, AbdelMalik P (2009). Evaluating Predictors of Geographic Area Population Size Cut-offs to Manage Re-identification Risk. J Am Med Inform Assoc.

[B48] Panel on Confidentiality Issues Arising from the Integration of Remotely Sensed and Self-Identifying Data, National Research Council (2007). Putting People on the Map: Protecting Confidentiality with Linked Social-Spatial Data.

[B49] Brownstein JS, Cassa CA, Mandl KD (2006). No place to hide–reverse identification of patients from published maps. N Engl J Med.

[B50] Google Street View. http://maps.google.com/help/maps/streetview/.

[B51] Kamel Boulos MN, Scotch M, Cheung KH, Burden D (2008). Web GIS in practice VI: a demo playlist of geo-mashups for public health neogeographers. Int J Health Geogr.

[B52] Tondel M, Axelson O (1999). Concerns about privacy in research may be exaggerated. BMJ.

