# Supplementary material for: Musings on privacy issues in health research involving disaggregate geographic data about individuals
Source: Int J Health Geogr. 2009 Jul 20;8:46. doi: 10.1186/1476-072X-8-46 (PMC2716332; doi:10.1186/1476-072X-8-46)
Supplement: Additional file 1 — A brief compilation and comparison of relevant personal information and privacy legislation in Canada and the UK, with particular focus on location and public health [file 1476-072X-8-46-S1.pdf]

A brief compilation and comparison of relevant personal information and privacy legislation in Canada and the UK, with particular focus on location and public health

Philip AbdelMalik - philip.abdelmalik@plymouth.ac.uk

A discussion on location privacy solutions for health research would be incomplete without reflecting on some of the underlying reasons that necessitate their development. The very notion of privacy is itself a complex fabric of interwoven philosophical and psychosocial threads. Perhaps this is why the associated bureaucratic and legal landscape is as complex as it is – and often blamed for the issue. A large majority of public health professionals consider privacy to be an obstacle to public health; when asked for the underlying reasons, survey respondents in Canada and the UK most commonly identified bureaucracy and legislation [1].

There is no universal legislation to guide and govern the activities of public health professionals, particularly where issues of privacy are concerned. Instead, nations have their own constraining or enabling privacy and data protection laws, with some being such a maze of cross-referenced "legalese" that familiarising oneself with them – let alone gaining a thorough understanding of them – becomes a daunting task. What ensues is a brief compilation and comparison of relevant personal information and privacy legislation in Canada and the United Kingdom (UK), with particular focus on location and public health as seen and understood by an epidemiologist.

Overview

The Canadian privacy-legislation landscape is additionally muddled by its political system: ten provinces and three territories, each with its own legislation and jurisdiction over its own health system. Overarching is the federal government, providing guidelines, support, oversight and funding. Although the words "privacy" and "personal information" do not occur anywhere in Canada's Constitution (Charter of Rights and Freedoms) [2], Section 7, granting the right to life, liberty and security, and Section 8, guaranteeing protection from unreasonable search and seizure, have been determined by the courts to capture the right to privacy [3,4]. These cases have expanded on the Charter sections to include privacy as related to protection from government or other intrusion, autonomy, and dignity.

Federally, Canada has two privacy laws. The *Privacy Act* [5] governs roughly 160 federal public bodies, whereas the *Personal Information and Protection of Electronic Documents Act* (PIPEDA) [6] governs private sector organisations regulated federally and provincially. Provinces with privacy legislation similar to *PIPEDA* are exempt from its provincial aspect. At the time of writing, British Columbia, Alberta and Québec have such legislation, and Ontario has health-specific legislation that exempts it from the corresponding section.

All provinces and territories have legislation similar to the *Privacy Act*, whereas only three provinces have private-sector legislation similar to *PIPEDA*. In addition, four provinces have specific health *information* legislation: Alberta, Manitoba, Ontario and Saskatchewan.

The UK has three legal jurisdictions: England and Wales, Scotland and Northern Ireland. However, it itself is also part of a larger community - the European Union (EU). European Union legislation is generally intended to "direct" that of its member states, and takes precedence in cases where there is no concurrence; the UK is obligated to align itself with EU law (referred to as Community law) [7] or else give way in a court of law to the latter [8]. Let us therefore begin with the EU.

The concepts of privacy and personal information are captured in core EU legislative documents as fundamental rights. The *European Convention for the Protection of Human Rights and Fundamental Freedoms* (*ECHR*), building on the 1948 *Universal Declaration of Human Rights* [9], includes a "Right to respect for private and family life" in Article 8 [10]. The *Charter of Fundamental Rights of the European Union*, proclaimed in 2000, builds on the ECHR [11]. Updated in 2007, the Charter includes two particularly relevant articles. Article 7 reiterates the *ECHR*'s position on the respect for private and family

life, whereas Article 8 explicitly limits the processing of personal data to specified purposes, requiring either individual consent or legislated “permission”.

Recognising the importance of data sharing and the threats and benefits of developing technologies, the EU introduced a number of legislative pieces to harmonise, regulate and facilitate the flow of personal information. In 1995, *Directive 95/46/EC* was adopted for the protection of personal data [12] - the core directive at the heart of data protection in EU member states. It does not, however, apply, to personal information used solely for personal reasons, household activities, public security, national defence or criminal law enforcement, and falls short when dealing with issues around communication. Two years later, the EU adopted *Directive 97/66/EC* for protecting privacy and confidentiality in telecommunications [13]. As technology and the web became increasingly ubiquitous, this directive quickly became limited in scope. It was therefore replaced in 2002 by *Directive 2002/58/EC* [14] covering electronic communications more broadly, and updated again in 2006 by *Directive 2006/24/EC* [15]. In addition, *Data Protection Regulation (EC) 45/2001* [16] ensures the protection of personal information in EU institutions and bodies, such as the European Parliament, for example, and accountability to a governing body, the European Data Protection Supervisor.

In the UK, the *Data Protection Act* was first enacted on July 12, 1984, thereby preceding the *Directive on Data Protection* adopted by the European Union (EU) by more than a decade. Upon adoption of the EU directive, however, the Act was amended in 1998. Though simpler than Canadian legislation in the sense that it applies to both public and private entities, it is none-the-less a complex document. In 2003, Lord Phillips of the Supreme Court of Judicature, Court of Appeal (Civil Division) in the UK referred to it as “…a cumbersome and inelegant piece of legislation” [17]. Other UK health-related Acts have been amended to reference the *Data Protection Act 1998*, including the *Access to Health Records Act 1990*, the *Access to Medical Reports Act 1988* and the *Access to Personal Files and Medical Reports (Northern Ireland)*. The UK also has a *Health and Social Care Act 2008* [18], which replaced its 2001 predecessor and legislated the creation of a Care Quality Commission for the protection and promotion of the health, safety and welfare of the public. The Act makes it an offence to recklessly disclose confidential personal information obtained by the Commission that “relates to and identifies an individual.” (S. 76)

Scotland has a *Freedom of Information Act 2002*, but a search on the UK Office of Public Sector Information website [19] yielded no specific data protection legislation for either Scotland or Northern Ireland. Scotland also has a *Public Health Act* enacted just last year, in 2008 [20], which obligates Scottish Ministers, health boards and local authorities to protect public health. It allows for the disclosure of information to facilitate its directives despite any other legal prohibition or restriction, except, interestingly, the *Data Protection Act 1998* (S. 117(6)). Northern Ireland's *Health and Social Care (Reform) Act 2009* [21] has a similar clause (S. 13(8)).

Both Canada and the UK have a tapestry of legislative documents in place to protect the privacy of personal information “…as something worth protecting as an aspect of human autonomy and dignity.” [22] But what, exactly, constitutes personal information?

Definitions

There is no consistent definition for “personal information” in Canadian legislation. Where a definition is included, it ranges from “information about an identifiable individual” in Alberta’s *Personal Information Protection Act* [23] to very well-defined and explicit components in Manitoba’s *Freedom of Information and Protection of Privacy Act* [24]. Of the 30 acts and regulations reviewed, four include health information in their definition of personal information, three include location information, 14 include both and nine include neither (Table 1).

This definition of personal information as pertaining to an “identifiable individual” appears quite often in legislation, including in *Directive 95/46/EC*. However, the *Directive* goes one step further to clarify: “…an identifiable person is one who can be identified, directly or indirectly, in particular by reference to an identification number or to one or more factors specific to his physical, physiological, mental, economic, cultural or social identity" [12]. Health information is defined as a “special” category of personal information (S. III, Article 8 (1)), but there is no specific mention of location information in the *Directive*.

In the UK, the *Data Protection Act 1998* defines "personal data" vaguely as any information that, in isolation or in concert with other data available to the data controller, can identify a living individual. The *Act* also includes health in the definition of "sensitive personal data", but does not capture location information specifically. As mentioned previously, the *Health and Social Care Act 2008* also identifies confidential personal information as that which “relates to and identifies and individual”, but does not specifically identify location as part of that definition.

As recent as April 2009, the Supreme Court of Canada stated that “Privacy analysis is laden with value judgements that are made from the independent perspective of the reasonable and informed person who is concerned about the long-term consequences of government action for the protection of privacy” [25]. As described, the definition of “personal information” in most cases casts a wide net, capturing anything and everything that can subjectively be argued as identifying. This has obvious implications on the use of disaggregate geographic data in health research. Or does it? The answer depends on the applications and exceptions made in the legislation.

Application and exceptions

Legislation in Canada, the EU and the UK specifically limits the processing of personal information. What constitutes “processing”, however, is not consistently defined across legislation. The broadest definition to capture what this means is found in EU *Directive 95/46/EC*: "any operation or set of operations which is performed upon personal data, whether or not by automatic means, such as collection, recording, organisation, storage, adaptation or alteration, retrieval, consultation, use, disclosure by transmission, dissemination or otherwise making available, alignment or combination, blocking, erasure or destruction". Generally, any such processing of personal information is prohibited *in the absence of the individual’s informed consent*, unless it is first stripped of all identifying information (thereby ceasing to be personal information according to the legal definition).

In public health research, however, it is often impossible or impractical to pursue informed consent. Despite being incredibly information and data-rich, health researchers in both Canada and the UK have often expressed frustration over their inability to use existing data due to privacy concerns [1]. Is the prohibition based on the legislation?

Generally, in the absence of an individual's consent, the legislation does explicitly allow for some exceptions, particularly in the interests of national security. However, there is a lack of clarity and consistency, specifically around processing for public health purposes. Article 35 of the *Charter of Fundamental Rights of the European Union* emphasises the right to health care, and states “A high level of human health protection shall be ensured in the definition and implementation of all Union policies and activities” [11]. In almost all cases, exceptions are also made for research, as long as the individuals whose data is processed are not identified in the results. Generally, the individual whose information has been disclosed should be informed; however, provisions are also made for cases where doing so is impossible or unreasonable.

The decision around whether or not the processing of the information is permitted under these exceptions is somewhat vague and inconsistent. In Canada, for example, the four provinces with health information legislation delegate the decision making authority to research ethics boards; otherwise, it is generally delegated to the head of the data-holding organisation. In the case of EU institutions, processing is only permissible after consultation with the European Data Protection Supervisor [16], whereas the UK *Data Protection Act 1998* exception for research (S. 4(33)) is unclear as to the decision-making authority. This leads to issues around governance.

Governance

In Canada, the Office of the Privacy Commissioner (OPC) is responsible for protecting and promoting the privacy rights of Canadians by overseeing compliance with Canadian federal privacy legislation. Each province and territory also has its own privacy commissioners who oversee their respective jurisdictions. As previously noted, health information legislation in Alberta, Saskatchewan, Manitoba and Ontario also delegates decision-making authority on these matters to research ethics boards.

The EU, as previously mentioned, has established the office of the European Data Protection Supervisor [26] for oversight of EU institution activities. The UK's equivalent of Canada's Office of the Privacy Commissioner is the Information Commissioner's Office (ICO) [27]. The legislation does not specifically mention research ethics boards or committees, and is unclear as to decision-making authority – in most cases, it seems to lie with the data controllers.

Implications and final thoughts

The privacy of personal information is a recognised and important human right, protected through multiple intertwined acts and regulations in Canada, the EU and the UK. In the absence of informed consent, the legislation generally allows for the processing of an individual's personal information – which is any information that can identify the individual, and therefore includes health and disaggregate location information – for research purposes, subject to approval by the appropriate authority. However, guidelines are lacking, and authorities tend to err on the conservative side, resulting in much expressed frustration by health researchers. In the absence of frameworks to inform the processing of personal information, the only other alternative (besides seeking informed consent from every individual) for health researchers is the use of de-identification techniques, such as might be applied through privacy-preserving solutions involving disaggregate geographic data.

It has been suggested that privacy in the United States, Canada and the European Union have their bases in slightly different philosophical constructs: in the United States, privacy is anchored in protection from the government; in Canada, in principles of autonomy and control; and in the European Union, the focus is more on dignity and public image [28]. The argument is made that the Canadian model offers the appropriate "middle-ground" – after all, if individuals truly do have control over their own personal information, then they can choose to protect it from the government and others, and their dignity as far as public image is concerned is in their own hands. If we accept this definition of privacy – that is, having control over one's own personal information – then one might ask whether de-identification really solves the issue. Perhaps what is really needed is public health specific clarification in the legislation, public and practitioner education, and clear and concise frameworks and guidelines.

Public health practitioners around the world are increasingly recognising the importance of having some understanding of the legal system, and a working relationship with the legal profession [29]. Unfortunately, the relationship typically tends to be unidirectional. Just as privacy is a multifaceted and complex concept, so too is the required collaboration resulting from the interdependency of public health and legislation. And yet, the legal profession has not fully recognised the interdependence of the two fields [29]. While the privacy debate in public health may be fuelled in part by misperceptions of public health practitioners, it is very much coupled with a lack of understanding of the requirements of public health by legal practitioners. "Privacy laws are most burdensome and least effective when they apply broadly, without proper concern for the settings in which they operate, the types of information that they cover, the obligations that they impose and the purposes they were designed to serve" [30]. The issue can only be truly addressed through interdisciplinary collaboration. Until that happens, and until we recognise the importance and value of public health research and its implications on the health of individuals, we will continue to grapple with alternate de-identification solutions and sub-optimal data.

Table 1: Inclusion of health and location information in the definitions of "personal information" in Canadian legislation

Jurisdiction	Act	Reference	In Definition	
			Health	Location
Canada	The Privacy Act [5]	R.S.C. 1985, c. P-21	✓	✓
Canada	Personal Information Protection and Electronic Documents Act [6]	S.C. 2000, c. 5 P-8.6	✓	
B.C.	Freedom of Information and Protection of Privacy Act [31]	R.S.B.C. 1996, c. 165		
B.C.	Personal Information Protection Act [32]	S.B.C. 2003, c. 63		
B.C.	Freedom of Information and Protection of Privacy Regulation [33]	B.C. Reg 323/93		
B.C.	Personal Information Protection Act Regulations [34]	B.C. Reg. 473/2003		✓
B.C.	British Columbia Cancer Agency Research Information Regulation [35]	B.C. Reg. 286/91	✓	✓
B.C.	Privacy Act [36]	R.S.B.C. 1996, c. 373		
AB	Health Information Act [37]	R.S.A. 2000, c. H-5	✓	✓
AB	Freedom of Information and Protection of Privacy Act [38]	R.S.A. 2000, c. F-25	✓	✓
AB	Personal Information Protection Act [23]	S.A. 2003 c. P-6.5		
AB	Personal Information Protection Act Regulation [39]	AR 366/2003		✓
SK	The Health Information Protection Act [40]	S.S. 1999, c. H-0.021	✓	
SK	The Freedom of Information and Protection of Privacy Act [41]	SS. 1990-91, c. F-22.01		✓
SK	The Local Authority Freedom of Information and Protection of Privacy Act [42]	SS. 1990-91, c. L-27.1	✓	✓
MB	The Personal Health Information Act [43]	C.C.S.M., c. P-33.5	✓	
MB	The Freedom of Information and Protection of Privacy Act [24]	C.C.S.M., c. F-175	✓	✓
ON	Personal Health Information Protection Act [44]	S.O. 2004, c. 3	✓	
ON	Freedom of Information and Protection of Privacy Act [45]	R.S.O. 1990, c. F-31	✓	✓
ON	Municipal Freedom of Information and Protection of Privacy Act [46]	R.S.O. 1990, c. M.56	✓	✓
QC	An Act respecting Access to documents held by public bodies and the protection of personal information [47]	R.S.Q., c. A-2.1		
QC	An Act respecting the Protection of personal information in the private sector [48]	R.S.Q., c. P-39.1		
N.B.	Protection of Personal Information Act [49]	S.N.B. 1998, c. P-19.1		
N.S.	Freedom of Information and Protection of Privacy Act [50]	S.N.S. 1993, c. 5, s. 1	✓	✓
N.S.	Health Protection Act [51]	S.N.S. 2004, c. 4, s. 1		
P.E.I.	Freedom of Information and Protection of Privacy Act [52]	R.S.P.E.I. 1988, c. F-15.01	✓	✓
NL	Access to Information and Protection of Privacy Act [53]	S.N.L. 2002, c. A-1.1	✓	✓
YK	Access to Information and Protection of Privacy Act [54]	R.S.Y. 2002, c. 1	✓	✓
N.T.	Access to Information and Protection of Privacy Act [55]	S.N.W.T. 1994, c. 20	✓	✓
NU	Access to Information and Protection of Privacy Act [56]	S.N.W.T. 1994, c.20	✓	✓

References

1. AbdelMalik P, Kamel Boulos MN, Jones R: The perceived impact of location privacy: a web-based survey of public health perspectives and requirements in the UK and Canada. *BMC Public Health* (2008), 8:156. [http://www.biomedcentral.com/1471-2458/8/156]

2. Canadian Charter of Rights and Freedoms. (March 1982) [http://laws.justice.gc.ca/en/charter/1.html]

3. R. v. Morgentaler, [1988] 1 S.C.R. 30. Supreme Court of Canada: January 28, 1988 [http://scc.lexum.umontreal.ca/en/1988/1988rcs1-30/1988rcs1-30.html]

4. Hunter et al. v. Southam Inc., [1984] 2 S.C.R. 145. Supreme Court of Canada: September 17, 1984 [http://csc.lexum.umontreal.ca/en/1984/1984rcs2-145/1984rcs2-145.html]

5. The Privacy Act, R.S.C. 1985, c. P-21. (1985) [http://laws.justice.gc.ca/en/P-21/section-[section-no].html]

6. Personal Information Protection and Electronic Documents Act, S.C. 2000, c. 5 P-8.6. (2000) [http://laws.justice.gc.ca/en/P-8.6/section-[section-no].html]

7. European Communities Act 1972. (1972)

8. European Judicial Network in civil and commercial matters: Legal Order - England and Wales. Last Updated: 19-8-2004; Accessed: 5-7-2009 [http://ec.europa.eu/civiljustice/legal_order/legal_order_eng_en.htm]

9. Universal Declaration of Human Rights. (December 1948) [http://www.un.org/Overview/rights.html]

10. Convention for the Protection of Human Rights and Fundamental Freedoms as amended by Protocol No. 11. (September 2003) [http://www.echr.coe.int/NR/rdonlyres/D5CC24A7-DC13-4318-B457-5C9014916D7A/0/EnglishAnglais.pdf]

11. Charter of Fundamental Rights of the European Union (2000/C 364/01). (2000) [http://eur-lex.europa.eu/LexUriServ/LexUriServ.do?uri=OJ:C:2000:364:0001:0022:EN:PDF]

12. Directive 95/46/EC of the European Parliament and of the council of 24 October 1995 on the protection of individuals with regard to the processing of personal data and on the free movement of such data. (November 1995) [http://eur-lex.europa.eu/LexUriServ/LexUriServ.do?uri=CELEX:31995L0046:EN:HTML]

13. Directive 97/66/EC of the European Parliament and of the Council of 15 bDecember 1997 concerning the processing of personal data and the protection of privacy in the telecommunications sector. (January 1998) [http://eur-lex.europa.eu/LexUriServ/LexUriServ.do?uri=OJ:L:1998:024:0001:0008:EN:PDF]

14. Directive 2002/58/EC of the European Parliament and of the Council of 12 July 2002 concerning the processing of personal data and the protection of privacy in the electronic communications sector (Directive on privacy and electronic communications). (July 2002) [http://eur-lex.europa.eu/LexUriServ/LexUriServ.do?uri=CELEX:32002L0058:EN:NOT]

15. Directive 2006/24/EC of the European Parliament and of the Council of 15 March 2006 on the retention of data generated or processed in connection with the provision of publicly available electronic communications services or of public communications networks and amending Directive 2002/58/EC. (May 2006) [http://eur-lex.europa.eu/LexUriServ/LexUriServ.do?uri=CELEX:32006L0024:EN:NOT]

16. Regulation (EC) No 45/2001 of the European Parliament and of the Council of 18 December 2000 on the protection of individuals with regard to the processing of personal data by the

Community institutions and bodies and on the free movement of such data. (January 2001) [http://eur-lex.europa.eu/LexUriServ/LexUriServ.do?uri=OJ:L:2001:008:0001:0022:EN:PDF]

17. Naomi Campbell and MGN Limited, [2002] EWCA Civ 1373. Supreme Court of Judicature, Court of Appeal (Civil Division): October 14, 2002 [http://www.bailii.org/ew/cases/EWCA/Civ/2002/1373.html]

18. Health and Social Care Act 2008 (c. 14). (2008) [http://www.opsi.gov.uk/acts/acts2008/ukpga_20080014_en_1]

19. Office of Public Sector Information. Last Updated: 16-6-2009; Accessed: 2009 [http://www.opsi.gov.uk/]

20. Public Health etc. (Scotland) Act 2008 (asp 5). (2008) [http://www.opsi.gov.uk/legislation/scotland/acts2008/asp_20080005_en_1]

21. Health and Social Care (Reform) Act (Northern Ireland) 2009 (c. 1). (2009) [http://www.opsi.gov.uk/legislation/northernireland/acts/acts2009/nia_20090001_en_1]

22. Campbell v. MGN Limited, [2004] UKHL 22. House of Lords Appellate Committee: May 6, 2004 [http://www.bailii.org/uk/cases/UKHL/2004/22.html]

23. Personal Information Protection Act, S.A. 2003, c. P-6.5. (2003) [http://www.canlii.org/en/ab/laws/stat/sa-2003-c-p-6.5/latest]

24. Freedom of Information and Protection of Privacy Act, C.C.S.M. c. F-175. (1997) [http://www.canlii.org/mb/laws/sta/f-175/20090324/whole.html]

25. R. v. Patrick, [2009] S.C.C. 17. Supreme Court of Canada: April 9, 2009 [http://scc.lexum.umontreal.ca/en/2009/2009scc17/2009scc17.html]

26. European Data Protection Supervisor. Last Updated: 2009; Accessed: 2009 [http://www.edps.europa.eu/EDPSWEB/]

27. Information Commissioner's Office. Last Updated: 2009; Accessed: 2009 [http://www.ico.gov.uk/]

28. Levin A, Nicholson MJ: Privacy law in the United States, the EU and Canada: the allure of the middle ground. *University of Ottawa Law and Technology Journal* (2005), 2(2):357-395.

29. Hoffman RE, Lopez W, Matthews GW, Rothstein MA, Foster KL: Law in Public Health Practice. New York: Oxford University Press, 2007

30. Cate FH: Privacy in perspective. Washington, D.C.: The AEI Press, 2001

31. Freedom of Information and Protection of Privacy Act, R.S.B.C. 1996, c. 165. (1996) [http://www.qp.gov.bc.ca/statreg/stat/F/96165_01.htm]

32. Personal Information Protection Act, S.B.C. 2003, c. 63. (2003) [http://www.qp.gov.bc.ca/statreg/stat/P/03063_01.htm]

33. Freedom of Information and Protection of Privacy Regulation, B.C. Reg 323/93. (1993) [http://www.qp.gov.bc.ca/statreg/reg/F/323_93.htm]

34. Personal Information Protection Act Regulations, B.C. Reg. 473/2003. (2003) [http://www.qp.gov.bc.ca/statreg/reg/P/473_2003.htm]

35. British Columbia Cancer Agency Research Information Regulation, B.C. Reg. 286/91. (1991) [http://www.qp.gov.bc.ca/statreg/reg/h/health/286_91.htm]

36. Privacy Act, R.S.B.C. 1996, c. 373. (1996) [http://www.qp.gov.bc.ca/statreg/stat/P/96373_01.htm]

37. Health Information Act, R.S.A. 2000, C. H-5. (2000) [http://www.assembly.ab.ca/HIAReview/Health_Information_Act.pdf]

38. Freedom of Information and Protection of Privacy Act, R.S.A. 2000, c. F-25. (2000) [http://www.iijcan.org/ab/laws/sta/f-25/20080818/whole.html]

39. Personal Information Protection Act Regulation, AR 366/2003. (2003) [http://www.qp.gov.ab.ca/documents/Regs/2003_366.cfm?frm_isbn=0779725050]

40. Health Information Protection Act, S.S. 1999, c. H-0.021. (1999) [http://www.qp.gov.sk.ca/documents/english/Statutes/Statutes/H0-021.pdf]

41. The Freedom of Information and Protection of Privacy Act, SS. 1990-91, c. F-22.01. (1990) [http://www.qp.gov.sk.ca/documents/English/Statutes/Statutes/F22-01.pdf#]

42. The Local Authority Freedom of Information and Protection of Privacy Act, SS. 1990-91, c. L-27.1. (1990) [http://www.qp.gov.sk.ca/documents/English/Statutes/Statutes/L27-1.pdf]

43. The Personal Health Information Act, C.C.S.M. c. P-33.5. (1997) [http://web2.gov.mb.ca/laws/statutes/ccsm/p033-5e.php]

44. Personal Health Information Protection Act, S.O. 2004, c. 3. (2004) [http://www.e-laws.gov.on.ca/html/statutes/english/elaws_statutes_04p03_e.htm]

45. Freedom of Information and Protection of Privacy Act, R.S.O. 1990, c. F-31. (1990) [http://www.e-laws.gov.on.ca/html/statutes/english/elaws_statutes_90f31_e.htm]

46. Municipal Freedom of Information and Protection of Privacy Act, R.S.O. 1990, c. M.56. (1990) [http://www.e-laws.gov.on.ca/html/statutes/english/elaws_statutes_90m56_e.htm]

47. An Act respecting access to documents held by public bodies and the protection of personal information, R.S.Q., c. A-2.1. (1982) [http://www2.publicationsduquebec.gouv.qc.ca/dynamicSearch/telecharge.php?type=2&file=/A_2_1/A2_1_A.html]

48. An Act respecting the protection of personal information in the private sector, R.S.Q., c. P-39.1. (1993) [http://www2.publicationsduquebec.gouv.qc.ca/dynamicSearch/telecharge.php?type=2&file=/P_39_1/P39_1_A.html]

49. Protection of Personal Information Act, S.N.B. 1998, c. P-19.1. (1998) [http://www.gnb.ca/acts/acts/p-19-1.htm]

50. Freedom of Information and Protection of Privacy Act, S.N.S. 1993, c. 5, s. 1. (1993) [http://www.gov.ns.ca/legislature/legc/statutes/freedom.htm]

51. Health Protection Act, S.N.S. 2004, c. 4, s. 1. (2004) [http://www.gov.ns.ca/legislature/legc/statutes/healthpr.htm]

52. Freedom of Information and Protection of Privacy Act, R.S.P.E.I. 1988, c. F-15.01. (1988) [http://www.gov.pe.ca/law/statutes/pdf/f-15_01.pdf]

53. Access to Information and Protection of Privacy Act, S.N.L. 2002, c. A-1.1. (2002) [http://www.assembly.nl.ca/legislation/sr/statutes/a01-1.htm]

54. Access to Information and Protection of Privacy Act, R.S.Y. 2002, c. 1. (2002) [http://www.gov.yk.ca/legislation/acts/atipp.pdf]

55. Access to Information and Protection of Privacy Act, S.N.W.T. 1994, c. 20. (1994) [http://www.justice.gov.nt.ca/pdf/ACTS/Access_to_Information.pdf]

56. Access to Information and Protection of Privacy Act, S.N.W.T. 1994, c. 20. (1994) [http://action.attavik.ca/home/justice-gn/attach-en_conlaw_prediv/Type002.pdf]
